# Severe cholestatic jaundice after a single administration of ajmaline; a case report and review of the literature

**DOI:** 10.1186/1471-230X-14-60

**Published:** 2014-04-02

**Authors:** Benjamin H Mullish, Rishi K Fofaria, Belinda C Smith, Kirsty Lloyd, Josephine Lloyd, Robert D Goldin, Ameet Dhar

**Affiliations:** 1 Section of Hepatology, Department of Medicine, St Mary’s Hospital, Imperial College London, London, UK; 2 Department of Histopathology, St Mary’s Hospital, Imperial College London, London, UK; 3 Section of Hepatology and Gastroenterology, Imperial College London, St Mary’s Hospital Campus, 10th floor, QEQM building, South Wharf Road, Paddington, London, UK

**Keywords:** Ajmaline, Drug-induced liver injury, Brugada syndrome, Liver, Pathology

## Abstract

**Background:**

Ajmaline is a pharmaceutical agent now administered globally for a variety of indications, particularly investigation of suspected Brugada syndrome. There have been previous reports suggesting that repetitive use of this agent may cause severe liver injury, but little evidence exists demonstrating the same effect after only a single administration.

**Case presentation:**

A 33-year-old man of Libyan origin with no significant past medical history underwent an ajmaline provocation test for investigation of suspected Brugada syndrome. Three weeks later, he presented with painless cholestatic jaundice which peaked in severity at eleven weeks after the test. Blood tests confirmed no evidence of autoimmune or viral liver disease, whilst imaging confirmed the absence of biliary tract obstruction. A liver biopsy demonstrated centrilobular cholestasis and focal rosetting of hepatocytes, consistent with a cholestatic drug reaction. Over the course of the next few months, he began to improve clinically and biochemically, with complete resolution by one year post-exposure.

**Conclusion:**

Whilst ajmaline-related hepatotoxicity was well-recognised in the era in which the drug was administered as a regular medication, clinicians should be aware that ajmaline may induce severe cholestatic jaundice even after a single dose administration.

## Background

Ajmaline is an alkaloid agent that was first isolated from the roots of *Rauwolfia serpentine*[1], and is classified as a class Ia antiarrhythmic. It has a number of different clinical indications for its use, with the major one at present being to elicit the characteristic ST segment changes on electrocardiogram (ECG) in patients suspected of having type 1 Brugada syndrome [2]. It has been used previously as maintenance treatment for different arrhythmias including atrial fibrillation in patients with Wolff-Parkinson-White syndrome and also in patients with ventricular tachycardia [3]. These, however, are now relatively rare indications for its use.

Although ajmaline administration appears to be generally safe, there have been previous reports of apparent drug-induced liver injury (DILI) related to its use. Some of these reports, however, were hampered by methodological flaws, and many of them were described in the era prior to easily-accessible serological confirmation/ exclusion of other liver insults, e.g. chronic viral hepatitis. Furthermore, there is to date only very scant evidence of ajmaline causing liver injury after only a single administration, the predominant means in which the drug is delivered at present. We here present an unusual case of severe cholestatic jaundice occurring after only a single administration of ajmaline.

## Case presentation

A 33-year-old man of Libyan origin was seen in cardiology clinic for investigation of pre-syncope. He had a strong family history of premature sudden death of unclear aetiology. As part of his assessment, he underwent an ajmaline provocation test, during which he had continuous ECG monitoring. After administration of 80 mg (equivalent to 1 mg/kg) of ajmaline, his monitoring demonstrated 2:1 atrioventicular block before reversion back to normal sinus rhythm. There had been no electrophysiological evidence of a type 1 Brugada syndrome prior to the assessment being stopped. The man was asymptomatic and had no evidence of arrhythmia during three hours of subsequent observation, and was discharged home.

Three weeks later, the man presented with painless jaundice, pruritus, nausea, dark urine and pale-coloured stools to his family physician. He had no personal or family history of liver disease and had not been jaundiced previously. He took no regular medications, and denied the use of alcohol, recreational drugs or herbal/ traditional therapies. Examination revealed jaundice and excoriation, but neither hepatomegaly nor features of chronic liver disease. An ultrasound of his abdomen demonstrated a normal appearance to the liver parenchyma, with no biliary dilatation. The common bile duct (CBD) measured 5 mm in maximal diameter, and no gallstones were seen. There was normal portal and hepatic venous flow. Sonographic appearances of the pancreas, spleen and kidneys were normal. He was kept under review.

**Figure 1:**
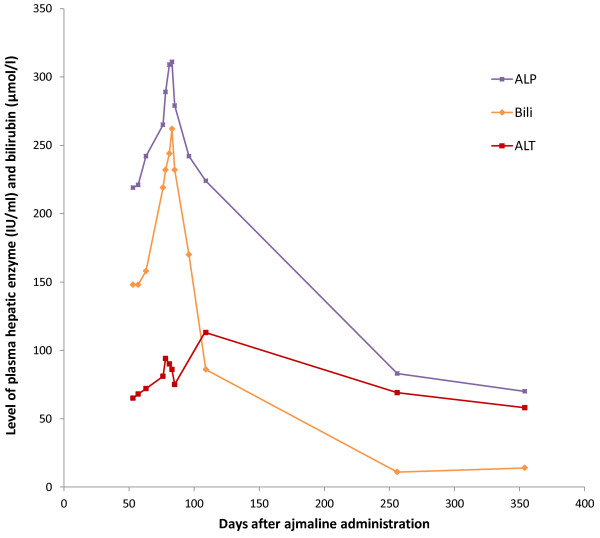
**Summary of the patient’s laboratory data during the course of the illness, including plasma liver enzymes and plasma bilirubin.** Time point zero is equivalent to the date that ajmaline was given intravenously. Abbreviations: ALT: Alanine aminotransferase; ALP: Alkaline phosphatase; Bili: Bilirubin.

**Figure 2:**
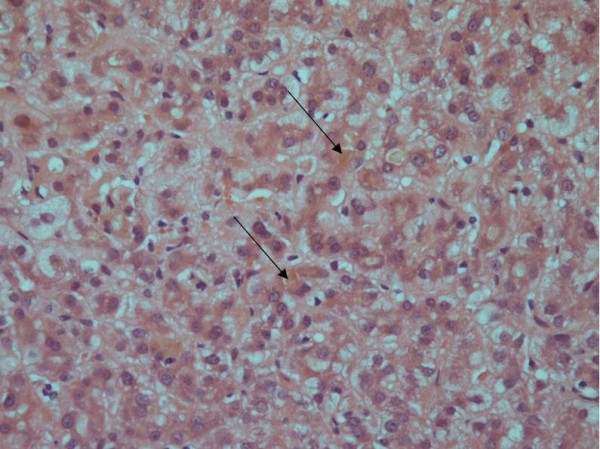
**Liver biopsy from the patient.** Marked centrilobular cholestasis is present, with otherwise normal bile duct structure and no evidence of hepatic fibrosis. Arrows indicate areas of cholestasis.

Ten weeks after the test, his jaundice and pruritus had worsened, and he was admitted to hospital for investigation. His liver enzyme profile was abnormal on admission and continued to deteriorate, peaking at day 81 after the administration of ajmaline with ALT 90 U/L, AST 67 U/L, ALP 309 U/L, GGT 120 U/L, and bilirubin 244 μmol/L (see Figure 1); no biochemistry results from prior to his illness were available. His platelet count, eosinophil count, prothrombin time and plasma albumin remained within normal limits throughout. His hepatitis A, B, C and E serology were negative. An auto-antibody profile (including anti-mitochondrial antibody, anti-smooth muscle antibody, anti-liver-kidney-muscle antibody, anti-nuclear and anti-nuclear cytoplasmic antibodies) was negative, and his serum markers of iron and copper metabolism normal. Inflammatory markers and white cell count differentials were normal, excluding underlying sepsis. A repeat ultrasound of his liver and biliary tree showed similar findings to that performed previously. Magnetic resonance cholangio-pancreatography (MRCP) confirmed a CBD of normal calibre, with no filling defects detected at any point in the biliary system. The patient underwent liver biopsy for further assessment. This demonstrated normal bile ducts, but striking centrilobular cholestasis and focal rosetting of hepatocytes (Figure 2). The biopsy results were consistent with a cholestatic drug reaction. Of note, the history of ajmaline administration had not been volunteered by the patient prior to the liver biopsy, and only emerged subsequently after liaison between the patient’s hepatologists and his family physician.

By nine days into his admission, his symptoms had begun to improve and his biochemistry stabilised, and he was discharged back to community care. When reviewed three weeks later, his pruritus had completely resolved. By one year after the initial exposure to ajmaline, his serum bilirubin and liver enzymes had near normalised (Figure 1).

## Conclusion

There is strong evidence that this man’s illness represented the unusual syndrome of ajmaline-related cholestatic jaundice. On the CIOS/RUCAM scale, a validated scoring system for suspected drug-induced liver injury, this patient scores 8, which is interpreted as ‘probable’ DILI [4,5]. This in conjunction with his histological findings indicates that it is very likely this patient’s liver injury was entirely ajmaline-induced, especially given the thorough exclusion of other possible aetiologies.

Ajmaline-related liver injury, at the time of writing this article, is not listed on the LiverTox website (http://www.livertox.nih.gov) that has been recently founded by the Liver Disease Research Branch of the National Institute of Diabetes and Digestive and Kidney Diseases (NIDDK) in collaboration with the National Library of Medicine (NLM) [6]. Case studies have described an association between repetitive ajmaline administration and liver injury, with the typical pattern identified being that of an initial acute hepatitis followed by a prolonged period of cholestasis [7-9]. However, no previous histologically confirmed cases of DILI following a single dose administration of ajmaline have been described in the literature. A single case report of cholestatic jaundice after a single administration of the medication has been described [10], but in this report biliary obstruction was not formally excluded via magnetic resonance imaging and the histological nature of the underlying injury was not delineated and hence not definitively confirmed to be secondary to this agent. This case describes a 54-year-old woman who presented with cholestatic jaundice three weeks after an ajmaline challenge, with a complete clinical and biochemical recovery at four months post-exposure. The time course and pattern of liver enzymes between the administration of the drug, the onset of jaundice and the recovery between our patient and the patient in the prior case report are similar.

Research into possible mechanisms of ajmaline-related cholestasis is limited, although immune precipitates within bile canaliculi have been found on liver biopsy samples taken from a patient with cholestasis after repetitive ajmaline exposure [8]. This may be consistent with ajmaline acting as a hapten when bound to proteins within the bile canaliculi. There is now evidence that an individual’s susceptibility to an idiosyncratic drug-induced liver injury may relate to a number of factors, including single nucleotide polymorphisms within particular HLA subtypes [11-13]. Ajmaline is metabolised by the cytochrome P450 enzyme 2D6. It is recognised that 71% of European Caucasians have functional alleles of this enzyme, whilst this is only the case in approximately 50% of Asians and Africans [14]. It may be the case in this patient that exposure to ajmaline in the context of a non-functional CYP2D6 allele, plus inheritance of a vulnerable HLA subtype, increased this patient’s propensity to drug-related liver injury.

In conclusion, hepatologists, pathologists, toxicologists and cardiologists should recognise the association between even a single administration of ajmaline and prolonged cholestatic jaundice with the presence of centrilobular cholestasis histologically. This case also highlights the importance of taking an accurate and thorough medication history from both the patient and his primary physician, including single-dose agents that patients may have been administered. It may be that patients from Africa and Asia have an increased propensity to this form of liver injury. Further research is needed to try and elucidate the mechanistic pathway by which ajmaline results in cholestasis.

### Consent

Written informed consent was obtained from the patient for publication of this Case report and any accompanying images. A copy of the written consent is available for review by the Editor of this journal.

## Competing interests

The authors declare that they have no competing interests.

## Authors’ contributions

BM, RKF, BCS and AD all contributed to writing the manuscript text. KL, JL and RDG were involved in the histological analysis of the liver biopsy. All authors read and approved the final manuscript.

## Pre-publication history

The pre-publication history for this paper can be accessed here:

http://www.biomedcentral.com/1471-230X/14/60/prepub
